# Jottings

**Published:** 1895-04

**Authors:** C. Carleton Smith

**Affiliations:** Philadelphia, Pa.


### JOTTINGS.

C. Carleton Smith, M. D., Philadelphia, Pa.

I have been in the habit for many years of jotting down for future reference characteristic symptoms of drugs which have been brought to my attention through reading medical literature. Having found many of these peculiar symptoms or key-notes very useful in my practice, I herewith lay before the readers of The Homœopathic Physician those which have proved reliable. It must, however, be borne in mind that key-notes—so named by my departed friend, Dr. Henry N. Guernsey—are not of themselves positive guides to the selection of the simillimum in a given case, but can be made use of simply as indices to such. To attempt to prescribe homœopathically on key-notes alone will, except in very rare instances, be sure to end in failure. For instance, I was on one occasion called to a case of strangury in an old lady, whom I found resting on her hands and knees, with head pressed to the floor, in order to pass her urine, while the sweat stood from every pore with the agony she was enduring. On the strength of the above key-note, which was revealed to me as I entered her room, I foolishly prescribed "Pariera-brava" as a snap-shot, expecting therefrom prompt relief. But I was doomed to disappointment, for I found at my next visit that the drug had most signally failed to bring to the sufferer the slightest relief. I then sat down, and took a picture of the whole case, just as the patient gave it to me. This picture said Cantharis, which I prescribed in the CM potency (Fincke), and the whole trouble promptly vanished. This case was so bad in all its aspects that I thought the old lady would surely die from exhaustion. But she did not do it. Nor has there been any return of the attack, now nearly two years.

I might add just here that the presence of strongly-smelling ammoniacal urine in these intensely acute attacks of strangury will be a guide in the selection of Pariera-brava, when we are hesitating between it, Berberis, and Cantharis.

EUGENIA-IAMBOS.—Lachrymation coming on in the evening and lasting into the night with a sensation as if fire were pouring out of the eyes.

Yellowish-bloody mucus in the mouth after dinner.

Pain in the os hyoides when swallowing.

Nausea, going off by smoking.

Scanty papescent *granular* stool after much straining, followed by spasmodic closing of the anus. (See, also, Alumina and Sulphur.)

Several sputtering fœtid discharges with burning in the abdomen.

Constant hawking up of bloody-yellow mucus.

Nightly cramps in soles of feet. Rhagades between the toes.

EUPHORBIA-OFFICINALIS.—Pale-red inflammation of the eyelids. Everything seems larger than it really is. Even in walking he raises his legs more than is necessary, because he imagines he is stepping over elevations. (See Amanita.)

Its effects upon the skin of head and face much resembles that of Rhus-tox.

Burning in throat and stomach as if a flame were rushing out.

Taste in mouth as if lined with rancid grease.

Great hunger, the stomach hanging down relaxed, causing a decided hollow feeling.

FERRUM-ACETICUM.—Copious expectoration of pus of a putrid taste, early in the morning. Also greenish pus. Slow, difficult asthmatic breathing, made better by walking and talking, or by constant reading or writing; worse when sitting still, and most violent when lying down, especially in evening.

Varicose veins of feet.

Burning, painful soreness of the back of the thumbs and toes from the slightest pressure.

FERRUM-MAGNETICUM.—This drug produces small warts on the hands. (See, also, Dulc.)

FERRUM-MUR.—Deposits of bright red crystals in urine.

FLUORIC-ACID.—Excessive ill-humor, quarrelling with every one in the house.

Dr. Hering, with two doses, cured an old invalid lady who quarrelled continually with all her nurses and relatives.

Sensation as if the eyelids were opened by force and a fresh wind was blowing upon them.

Clearness of sight and increased power of vision. The circle of vision seems to be greatly enlarged, so that he found luxurious enjoyment in looking at the same things he is used to see every day.

His teeth feel warm.

Several small round blood vesicles appear, of a bright-red color, like carmine, soft and compressible, resembling flesh warts. Look like enlargement of the capillaries.

Sensation as if burning vapor was emitted from the pores of the whole body.

Itching of all the cicatrices in the evening. All the cicatrices dating thirty-two years back are red around the edges.

GRAPHITES.—Urinous odor from the mouth and nose. Constant spasm in throat, obliging him to swallow as if he were choking, and food would not go down. Hard, knotty stools, the lumps united by mucous threads.

During menstruation, hoarseness, violent coryza, and catarrhal fever. Also dry cough and profuse sweats.

Morning sickness, swollen feet. (See, also, Lycopod.)

Swelling of prepuce, forming a large blister containing serum.

Itching inside of scrotum; darting pain in left spermatic cord.

Violent burning of eyes, as if some acrid matter was in them.

Can't bear the light of day, but can that of candlelight.

Black sweaty pores on the nose. (See, also, Sulphur and Digitalis.)

Smell in nose as of soot burning.

